# Effect of Blanching Plus Fermentation on Selected Functional Properties of Mealworm (*Tenebrio molitor*) Powders

**DOI:** 10.3390/foods9070917

**Published:** 2020-07-12

**Authors:** An Borremans, Sara Bußler, Sorel Tchewonpi Sagu, Harshadrai Rawel, Oliver K. Schlüter, Van Campenhout Leen

**Affiliations:** 1 Department of Microbial and Molecular Systems (M2S), Faculty of Engineering Technology, KU Leuven, Lab4Food, Campus Geel, B-2440 Geel, Belgium; an.borremans@kuleuven.be; 2 Leibniz Institute for Agricultural Engineering and Bioeconomy (ATB), Quality and Safety of Food and Feed, Max-Eyth-Allee 100, 14469 Potsdam, Germany; sbussler@atb-potsdam.de (S.B.); oschlueter@atb-potsdam.de (O.K.S.); 3 Institute of Nutritional Science, University of Potsdam, Arthur-Scheunert-Allee 114–116, D-14558 Nuthetal, Germany; sorelsagu@uni-potsdam.de (S.T.S.); rawel@uni-potsdam.de (H.R.)

**Keywords:** mealworm, fermentation, functional properties, insect proteins, SDS-PAGE

## Abstract

The aim of this study was to determine the effect of blanching followed by fermentation of mealworms (*Tenebrio molitor*) with commercial meat starter cultures on the functional properties of powders produced from the larvae. Full fat and defatted powder samples were prepared from non-fermented and fermented mealworm pastes. Then the crude protein, crude fat, and dry matter contents, pH, bulk density, colour, water and oil binding capacity, foaming capacity and stability, emulsion capacity and stability, protein solubility, quantity of free amino groups, and protein composition of the powders were evaluated. Regardless of the starter culture used, the blanching plus fermentation process reduced the crude and soluble protein contents of the full fat powders and in general impaired their water and oil binding, foaming, and emulsifying properties. Defatting of the powders improved most functional properties studied. The o-phthaldialdehyde assay revealed that the amount of free amino groups was higher in the fermented powders while sodium dodecyl sulfate polyacrylamide gel electrophoresis demonstrated that the soluble proteins of the fermented powders were composed of molecules of lower molecular mass compared to non-fermented powders. As molecular sizes of the soluble proteins decreased, it was clear that the protein structure was also modified by the fermentation process, which in turn led to changes in functional properties. In general, it was concluded that fermentation of mealworms with blanching as a pre-treatment does not contribute to the functional properties studied in this work. Nevertheless, the results confirmed that the properties of non-fermented powders are comparable to other food protein sources.

## 1. Introduction

In recent years, there has been an increased interest in the use of edible insects for food applications. In particular, mealworms (*Tenebrio molitor*, Linnaeus (Coleoptera: Tenebrionidae)), preferably reared on organic side streams to support a circular economy [1], are gaining attention as an alternative protein source due their high protein level, good amino acid profiles, and high levels of unsaturated fatty acids, vitamins, and minerals [2]. Various technologies are used for the stabilisation of mealworms and processing them into foods, most of which are based on heat application (oven drying and boiling). It can be interesting, both cost-wise and protein property-wise, to apply processes that do not involve heat or less heat. Fermentation is a non-thermal process in which a food matrix is subjected to the action of microorganisms or enzymes so that desirable biochemical changes cause modification to the product [3]. These modifications may result in modified sensory qualities, improved nutritional value, enhanced preservation, and/or increased economic value. In the study of Cho et al. [4], a liquid fermented seasoning was prepared using mealworms by applying the soy sauce fermentation process. Patrignani et al. [5] showed the great potential of *Yarrowia lipolytica* and *Debaryomyces hansenii* strains to produce cricket based food ingredients endowed with high food safety, functionality, sensory and technological properties. The strains increased the matrix digestibility and improved the sensory features of the raw material due to the production of many compounds characteristic of ripened and fermented foods. Fermentation of a mealworm paste has also been reported to be feasible with lactic acid starter cultures, as indicated by a rapid pH reduction [6,7]. During fermentation, some of the starter cultures tested generated free glutamic and aspartic acid. These two amino acids are responsible for the appreciated umami taste. The impact of fermentation on other properties of this paste has not been investigated so far and is the subject of this study. Fermentation is expected to alter the characteristics of the insect proteins, but it is not known how this translates into their nutritional value and technological functionality and hence in their application potential as food ingredients.

In Western countries, insects are believed to be better accepted by consumers when they are fragmented and included in a food as an ingredient, rather than in their whole form. For mealworms to be successful in food applications, they should ideally possess several desirable characteristics, referred to as functional properties. To date, only a few studies have considered the technical functionality of mealworm flours [8,9,10] or protein preparations thereof [10,11,12], such as solubility, water and oil binding, gelling, foaming, and emulsifying capacity. In general, mealworms have been found to have high water and oil binding, good emulsion properties, but moderate to poor foam and gelling properties. Their proteins exhibit good solubility in the pH range of 2 to 12, making them a suitable candidate for many food applications. Despite several studies on the functional properties of mealworms, the effects of fermentation on these properties are unclear. Some properties may be affected by fermentation as fermentation tends to alter the structure of proteins, which are the main functional constituents in emulsions, foams, and gels [13].

The objective of this study was to investigate the impact of a combination of treatments of blanching followed by fermentation on the functional properties of mealworm powders. Previous research of Borremans et al. [14] showed blanching to be indispensable prior to fermentation, to avoid the (extensive) endogenous microbiota present on the mealworms to disturb the fermentation. Mealworm pastes were produced and, apart from the control samples, either fermented with the commercial meat starter culture Bactoferm^®^ F-LC or with the pure culture *Lactobacillus farciminis*. The ability of these starter cultures to ferment a mealworm paste produced at laboratory scale was demonstrated in previous work [7,14]. Full fat and defatted powders were produced from all pastes and characterised with respect to moisture content, crude protein and crude fat content, pH, bulk density, and colour. To evaluate the fermentation-induced effects on protein functionality, water and oil binding, foaming and emulsion properties as well as protein solubility (pH 2 to 10) of protein extracts recovered from defatted and full fat powders were analysed. In addition, the content of free amino groups and the molecular weight distribution of the water-soluble proteins were analysed, which may shed (some) light on the effect of fermentation on the protein properties.

## 2. Materials and Methods

### 2.1. Sample Preparation and Processing

**Figure 1:**
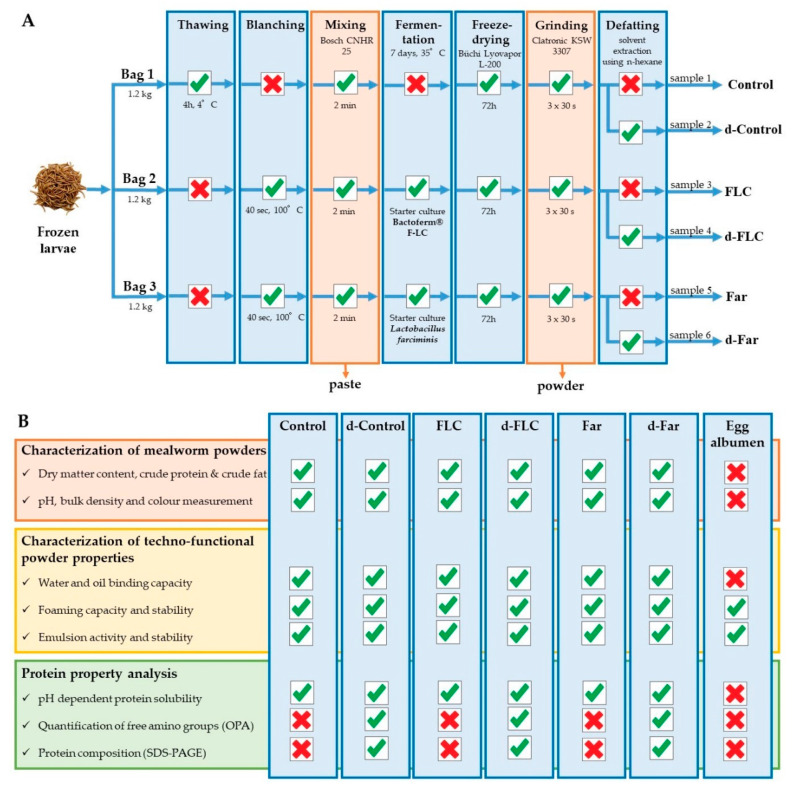
Schematic representation of sample preparation (**A**) and an overview of the performed analyses (**B**).

Mealworms were purchased from the commercial supplier Nusect (Ledegem, Belgium). The living mealworms were packaged in freezer bags (3 × 1.2 kg mealworms/bag, Euralpack NV, Schoten, Belgium), killed by freezing (−18 °C for at least 24 h) and stored frozen at −18 °C until further use. Mealworm powders (non-fermented, fermented and defatted samples) were prepared as shown in Figure 1A. Non-fermented samples were prepared by thawing one bag of mealworms for 4 h at 4 °C and mixing the larvae into a paste using a kitchen mixer as described earlier [7]. The larvae of the other two bags were blanched (40 s), mixed into a paste (2 min), triplicate volumes of 400 g were inoculated according to the manufacturers’ instructions with either the commercial meat starter culture Bactoferm^®^ F-LC (Chr. Hansen Holding A/S, Hoersholm, Denmark, 501091, mixture of *Staphylococcus xylosus*, *Lactobacillus curvatus* and *Pediococcus acidilactici*, 25 g/100 kg) or with the pure culture *Lactobacillus farciminis* (Chr. Hansen Holding A/S, Hoersholm, Denmark, 501167) to reach a level of ± 6.5 log cfu/g paste, and subsequently fermented according to Borremans et al. [7,14]. Briefly, 2.8% NaCl (*w*/*w*) and 0.75% d (+)-glucose (*w*/*w*) were added to the pastes to provide flavour, to control the background microbiota, and to fuel the fermentation as the fermentable sugar content of mealworms is low. After thorough mixing, the pastes were distributed over sterile 50 mL Falcon tubes (Sarstedt, Antwerp, Belgium) and incubated at 35 °C for seven days under normal atmospheric pressure conditions. Powders were produced from the non-fermented and fermented pastes by freeze drying (48 h, Büchi Lyovapor L-200, Büchi, Flawil, Switzerland) and grinding (60 s, Clatronic KSW 3307, Clatronic International GmbH, Kempen, Germany). To prepare defatted powders, solvent extraction using n-hexane was performed. A proportion of each powder was mixed with hexane (1:10 ratio, *v*/*w*) and stirred on a magnet stirrer for 1 h. After sedimentation of the solids, the hexane-fat-mixture was decanted and residual hexane was removed by evaporation overnight.

Figure 1B gives an overview of the performed analyses. The water and oil binding capacity of the powders were determined at their native pH, the foaming and emulsifying properties and the free amino groups at pH 7 (indicative of a neutralisation process), and protein solubility and protein composition were determined in the pH range of 2 to 12. Egg albumen powder served as reference material from Pulviver (Bastogne, Belgium).

### 2.2. Characterization of Mealworm Powders

#### 2.2.1. Crude Protein, Crude Fat and Dry Matter Content

Crude protein and crude fat contents were determined in triplicate as described by Bußler et al. [8]. Crude protein contents (NKjel, conversion factor 6.25) were determined using the method by Kjeldahl (Kjeldatherm Turbosog, Titrino plus 848, Gerhardt Analytical Systems, Königswinter, Germany), according to DIN EN 25663: Digestion and distillation (Kjeldahl Sampler System K- 370/371) were conducted as described by the Association of German Agricultural Investigation and Research Institutions [15]. Crude fat content of the flour fractions was determined according to the filter bag method Am 5-04 [16] as an indirect method for measuring crude oil (Filterbags XT4, ANKOM Technology, New York, NY, USA). Dry matter contents were determined by drying the mealworm powders in a forced-air oven (Memmert UFB500, Memmert GmbH, Schwabach, Germany) at 105 °C for 17 h.

#### 2.2.2. pH, Bulk Density and Colour Measurement

To determine pH, mealworm powders (3 × 1.0 g) were mixed with 10 mL of distilled water and vortexed for 30 s. The pH of the mixtures was measured using a pH meter (pHenomenal pH 1100H, VWR, Leuven, Belgium) with a SenTix 82 pH electrode (VWR). Bulk density was estimated by the modified method of Wang and Kinsella [17]. A 10.0 g sample was gently packed in a 100 mL graduated cylinder by tapping ten times on a bench top from a height of 5 cm. The final volume of the sample was recorded and the bulk density was expressed as g/mL of sample. Colour measurements were performed with a colorimeter using the CIElab scale (CR-5, Konica Minolta). Five independent measurements of a * (redness), b * (yellowness), and L * (lightness) parameters were carried out for triplicate samples of each powder type. Browning indices and colour difference (ΔE) were calculated according to the formula described by Lenaerts, Van Der Borght, Callens, and Van Campenhout [18], using non-fermented powder as reference.

### 2.3. Analysis of the Techno-Functional Properties of the Powders

#### 2.3.1. Water and Oil Binding Capacity

Water (WBC) and oil (OBC) binding capacity of the powders were determined in triplicate according to the method of Bußler et al. [8] with slight modifications. Briefly, 0.5 g of each powder was weighed into a pre-weighed centrifuge beaker to which 3.0 mL of distilled water or 3.0 mL of commercial rape seed oil had been added. The mixtures were stirred (60 s or two times 60 s for WBC and OBC, respectively) using a propeller stirrer and an overhead agitator (Yellowline^®^ OST Basic, IKA^®^, Wilmington, NC, USA), and centrifuged (Eppendorf 5810R, Eppendorf AG, Hamburg, Germany) at 3,900 *g* for 20 min. The samples were re-weighed after discarding the supernatant and putting the beakers upside-down on absorbent paper for 60 min. The differences in weight were calculated and the results were presented as gram of water or oil absorbed per gram of powder.

#### 2.3.2. Foaming Capacity and Stability

For foaming capacity and foaming stability determinations, 0.25% *w/v* protein suspensions were prepared at pH 7. Briefly, each sample (quantity depending on solubility of the proteins) was suspended in 100 mL distilled water and the pH was adjusted to 7 with 1.0 M NaOH or 1.0 M HCl. The solutions were stirred (INFORS HT labotron, MS-L GmbH, Wiesloch, Germany) for 30 min at room temperature and centrifuged (Eppendorf 5810R, Eppendorf AG, Hamburg, Germany) for 20 min (4 °C, 10,000 *g*). The supernatants were collected and stored at 4 °C until subsequent analyses. The foaming properties were determined fivefold by the method described by Zielińska et al. [10], with modifications. Twenty millilitre of supernatant was transferred into a 250 mL beaker and each sample was individually beaten in a high shear homogenizer mixer (16,000 rpm, 2 min, Ultra turrax, IKA, Staufen, Germany). The whipped sample was immediately transferred into a graduated cylinder and the total volume was read at time zero and 30 min after whipping. Foaming capacity (FC) and foaming stability (FS) were calculated using the formulas described by Zielińska et al. [10].

#### 2.3.3. Emulsion Capacity and Stability

Emulsifying properties were determined fivefold with the method of Zielińska et al. [10], with modifications. Protein solutions (0.25% *w/v*, pH 7) were prepared as described earlier and 10 mL of each solution was mixed in 50 mL tubes with an equal volume of rapeseed oil dyed with liquid natural carotene (M = 536.89 g/mol, Carl Roth, Karlsruhe, Germany). Following homogenization (20,000 rpm, 1 min, Ultra turrax, IKA, Staufen, Germany), the mixtures were centrifuged (Eppendorf 5810R, Eppendorf AG, Hamburg, Germany) at 3,000 *g* for 5 min and the volume of the individual layers were read. Emulsion stability was evaluated by heating the emulsion for 30 min at 80 °C. Then, the samples were centrifuged at 3,000 *g* for 5 min before the volumes of the individual layers were read. Emulsion capacity (EC) and emulsion stability (ES) were calculated using the formulas described by Zielińska et al. [10].

### 2.4. Analysis of the Protein Properties of the Powders

#### 2.4.1. pH Dependent Protein Solubility

Protein solubility was determined in the pH range of 2 to 12. Briefly, 0.1 g of each powder was mixed with 10 mL of distilled water and the pH of the mixture was adjusted to a value in the range of 2 to 12 using 1.0 M HCl or 1.0 M NaOH. The solutions were stirred on a rotary shaker (350 rpm) for 30 min and centrifuged (8,000× *g*, 20 min, 4 °C, Eppendorf 5417R, Eppendorf AG, Hamburg, Germany). The protein concentration of the supernatant was assessed by the Bradford method [19] using bovine serum albumin (Fluka, Buchs, Switserland) as a standard. The assay consisted of 800 µL of the protein extracts and 200 µL of Bradford reagent (Roti^®^-Quant, Carl Roth, Karlsruhe, Germany) reacted for 20 min at ambient temperature. The absorption maximum was analysed at 595 nm against a blank value (800 µL demineralized water, with a pH adjusted to the same pH of the sample, and 200 µL of Bradford reagent) using an UV/Vis spectrophotometer (BioPhotometer plus, Eppendorf, Hamburg, Germany). The dissolving procedure and spectrophotometric measurements were each performed in triplicate and the protein solubility was calculated according to the formula described by Zielińska et al. [10]. The remainder of the protein extracts was frozen at −18 °C for the quantification of free amino groups and the determination of the protein composition.

#### 2.4.2. Quantification of Free Amino Groups

To quantify the amount of free amino groups in the defatted mealworm powders, the o-phthaldialdehyde (OPA) assay was used. To this end, 100 µL of protein extract (pH 7, thawed at room temperature) was added to 800 µL OPA/NAC (N-acetyl-cysteine) reagent. This reagent was prepared by combining 1 mL OPA stock solution (34 mg OPA in 1 mL methanol), 25 mL of 0.3% *w/v* NAC (0.150 g NAC in 50 mL 200 mM borate buffer), 2.5 mL of 20% *w*/*w* SDS, and 21.5 mL distilled water. The mixtures were allowed to react at ambient temperature for 30 min before the absorbance was measured at 330 nm against a blank containing 100 µL of protein extract and 800 µL of the same OPA/NAC reagent but without OPA (the OPA stock solution was replaced by methanol). Three replicates of each measurement were included in the experiment and the free amino groups were calculated against an L-leucine (Merck KGaA, Darmstadt, Germany) standard curve. The results are expressed as millimolar free amino groups per gram of soluble protein.

#### 2.4.3. Protein Molecular Weight Distribution

Proteins of the defatted powders were analysed with sodium dodecyl sulphate polyacrylamide gel electrophoresis (SDS-PAGE) to determine their molecular weight distribution. Protein extracts (pH 2 to 12) were thawed at room temperature, sonicated (S10 Elmasonic, Elma Schmidbauer GmbH, Singen, Germany) for 5 min, and 500 μL of each replicate per pH-value was pooled. Electrophoresis was performed under reducing and denaturing conditions using Invitrogen NuPAGE 12% Bis-Tris precast protein gels with 12 wells (Thermo Fisher Scientific, Carlsbad, CA, USA). Non-Fermented samples were mixed with NuPAGE™ LDS Sample Buffer (containing glycerol, 2-mercaptoethanol, SDS, and Coomassie blue G250 at pH 8.4) at a ratio of 1:5, while the fermented samples were mixed at a ratio of 1:3 with the same sample buffer. As references, 10 mg of each powder from non-fermented and fermented samples was dissolved in 1500 µL sample buffer and diluted with sample buffer in a ratio of 1:2. After heating the mixtures at 100 °C for 5 min, samples were cooled to room temperature and each 15 μL of the reference and non-fermented samples and 10 μL of fermented samples were loaded onto the gel. An aliquot of 5 µL of Page Ruler Plus pre-stained broad range standard containing a nine protein ladder (protein composition in kDa of 10, 15, 25, 35, 55, 70, 100, 130, and 250; Thermo Fisher Scientific, Carlsbad, CA, USA) was loaded as well. Table S1 (Supporting information) presents the initial protein concentration and the protein quantities loaded. The separation was carried out under constant current (30 mA/Gel) for 120 min using NuPAGE MES SDS Running Buffer. After separation, the gels were stained overnight at room temperature with Coomassie blue solution (in 10% acetic acid) and then de-stained with 10% acetic acid for 3 h. The gels were scanned using Bio-5000 Professional VIS Gel Scanner (Provided by SERVA Electrophoresis GmbH, Heidelberg, Germany) and analysed with Image Lab Software (Bio-Rad Laboratories Ltd., Hemel Hempstead, UK). Electrophoresis experiments were repeated twice.

#### 2.4.4. Statistical Analysis

SPSS statistics (IBM SPSS Statistics version 25, New York, NY, USA) was used to statistically analyse the data generated. The data, reported as averages of at least three replicates, were subjected to one-way analysis of variances (ANOVA) to compare means. Next, Tukey’s post-hoc test was used to determine significant differences among samples. However, when variances were not equal, the Kruskal–Wallis test with the Dunn–Bonferroni post-hoc test was performed. For all tests a significance level of 0.05 was considered.

## 3. Results and Discussion

### 3.1. Characterization of Mealworm Powders

The dry matter, crude protein and crude fat contents of the different mealworm powders were determined and expressed on a dry matter basis (Table 1). The Control had a dry matter content of 96.26% and contained 49.68% of crude protein and 16.61% of crude fat. The protein and fat content were lower than the values reported for freeze-dried mealworms by Zhao et al. [11] (51.5% and 32.9%, respectively), Bußler et al. [8] (57.8% and 19.1%, respectively) and Lenaerts et al. [18] (59.96% and 28.35%, respectively). This heterogeneity in crude protein and crude fat content can be ascribed to differences in rearing and processing conditions as well as to differences in methods of analysis applied [20]. Blanching and fermentation with the starters Bactoferm^®^ F-LC and *L. farciminis* did not significantly influence the dry matter content of the full fat powders. The crude protein content, on the other hand, significantly (*p* < 0.05) decreased, while (concomitantly) the crude fat content significantly (*p* < 0.05) increased with blanching and fermentation. The literature reports various effects of fermentation on proteins in other food matrices. Several studies [21,22] observed an increase in pea and acha rice flour, respectively, while others [23,24] reported a decrease in crude protein content during fermentation of lupine and a melon type, respectively. An increase in crude protein content may be attributed to loss of dry matter (mainly carbohydrates). A decrease, on the other hand, is more difficult to explain, but can possibly be ascribed to a loss of (a small part of the) proteins during blanching by precipitation and/or the incorporation of NaCl and sugars during fermentation (lowering the relative content of nitrogen). Defatting of the mealworm powders (resulting in the powders d-Control, d-FLC and d-Far) contributed to a significant (*p* < 0.05) increase in the crude protein content of respectively 18.21%, 19.34%, and 17.31%. Subsequent analysis of the fat content revealed residual fat values of less than 5.34% for all samples.

Results of pH, bulk density and colour parameters are summarized in Table 1 as well. The pH of the Control was 6.28 and was unaffected by the defatting treatment. Fermentation significantly decreased (*p* < 0.05) the pH by the production of organic acids to values ranging from 4.56 to 4.68 for the full fat samples and from 4.70 to 4.77 for the defatted samples. Both hexane defatting as well as fermentation significantly increased (*p* < 0.05) the bulk density of all powders. The increase in bulk density (BD) by defatting may be caused by particles sticking to each other due to the presence of residual n-hexane, but the increase in BD by fermentation cannot be explained to date. According to Ogodo, Ugbogu, Onyeagba, and Okereke [25], the BD usually decreases during fermentation due to the breakdown of complex compounds into simpler molecules by microorganisms. As to the colour characteristics of the full fat powders, the fermented ones had a higher browning index and thus darker colour than the control powder. The subsequent defatting process noticeably decreased the browning index from ±70 to ±30 and eliminated statistical differences in colour between the powders. ΔE, the total colour difference between the fermented samples and the control powders, was recorded in the range of 1.16 to 2.55, which is considered to be noticeable by a human observer [26].

### 3.2. Impact of Fermentation on the Techno-Functional Properties of the Powders

#### 3.2.1. Water and Oil Binding Capacity

**Figure 2:**
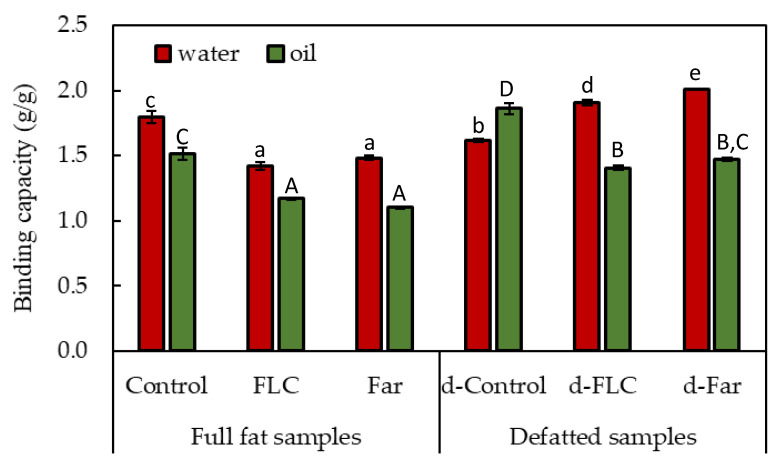
Water (WBC, red) and oil (OBC, green) binding capacity of full fat (Control, FLC and Far) and defatted (d-Control, d-FLC, d-Far) mealworm powders. Data are expressed as mean ± standard deviations (*n* = 3). Different letters (a,b,c,d,e for WBC and A,B,C,D for OBC) indicate significant (*p* < 0.05) differences between means.

WBC and OBC of the different mealworm powders are depicted in Figure 2. The WBC and OBC of the non-fermented and full fat samples was 1.79 ± 0.05 and 1.51 ± 0.05 g/g and it was 1.62 ± 0.01 and 1.86 ± 0.05 g/g in non-fermented, defatted samples. Bußler et al. [8] reported substantially lower WBC (0.8 g/g dry mass) and OBC (0.6 g/g dry mass) values for (non-fermented) full fat mealworm flour. Using a similar method of powder production to that used in this study, Zielińska et al. [10] reported a WBC of 1.29 g/g and an OBC of 1.71 g/g for ground mealworms. The different origin (likely including a different rearing method and/or substrate) of the mealworms as well as the different methods of analysis used can explain the variations in the results from different studies. Compared to other protein sources, the mealworm powders tested in this study had higher WBC and OBC values than those of soy flours (130% and 84%, respectively) and comparable WBC and OBC to those of egg white flours (168% and 135%, respectively [27]).

Irrespective of the starter cultures used, the blanching and fermentation process induced a significant decrease in WBC (from 1.9 to 1.4–1.5 g/g) and OBC (from 1.5 to 1.1–1.2 g/g) in the full fat samples. The lower protein contents of the fermented samples compared to those of non-fermented samples (Table 1), as well as changes in the quality of the proteins upon processing, can explain this reduction in WBC and OBC. Reduction in WBC by lactic acid fermentation was observed earlier for various flours, such as maize flour and sorghum flour [25]. Contradictory to the present study, the OBC of these flours was enhanced by fermentation with 1.2 and 0.8 mL/g, respectively. During fermentation, these proteins became partially unravelled and hence exposed buried hydrophobic groups that can bind more oil. Whereas blanching and fermentation decreased both WBC and OBC in powders that were not defatted afterwards, removing oil from the fermented mealworm powders significantly (*p* < 0.05) reduced OBC but increased WBC. Among all powders tested, the fermented and then defatted powders showed the highest WBC.

#### 3.2.2. Foaming Capacity and Stability

**Figure 3:**
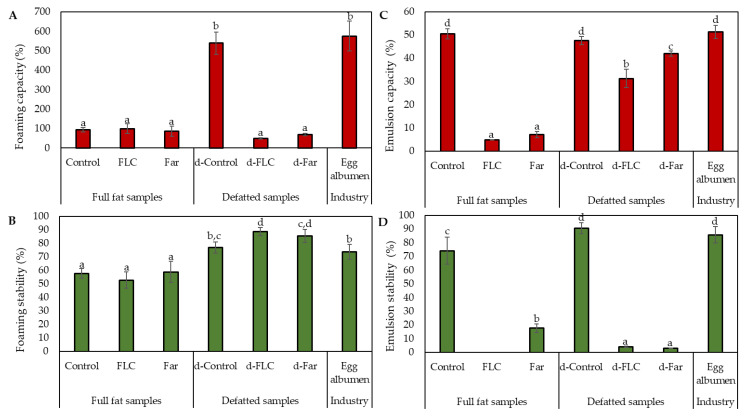
Foaming capacity (**A**), foaming stability (**B**), emulsion activity (**C**) and emulsion stability (**D**) of extracted protein solutions (0.25% *w*/*v*) from full fat (Control and the fermented samples FLC and FAR) and defatted (d-Control and the fermented samples d-FLC and d-Far) mealworm powders. Egg albumen solutions (0.25% *w*/*v*) were included as reference. Data are expressed as mean ± standard deviations (*n* = 5). Different letters (a,b,c,d) indicate significant (*p* < 0.05) differences between means.

Figure 3A,B describes the foaming capacity (FC) and foaming stability (FS) of the protein solutions (0.25% *w/v*, pH 7) extracted from the different mealworm powders. As a reference for FC and FS measurements, an egg albumen solution was used at the same concentration. Egg albumen has excellent foaming properties as it adsorbs rapidly on the air-liquid interface during whipping and rearranges to form a cohesive viscoelastic film via intermolecular interactions [28].

Figure 3A did not reveal any significant differences in FC among the full fat powders. The FCs ranged from 60 to 126% and were low when compared with the reference (575 ± 77%). Defatting markedly improved the FC of the control powder from 94 to 540%. This observation is consistent with the findings of Akpossan et al. [29] that the FCs of defatted flours were superior to those of full fat flours. In contrast to the Control, the FC of the fermented powders diminished upon defatting to 50 and 70%, respectively. These results demonstrate that the treatments used in this study (i.e., blanching and fermentation) may cause changes in the nature of the proteins, which lead to changes in foaming properties. The FS of the powders ranged from 47 to 92% (Figure 3B). Similar to the FC, results of FS showed no pronounced differences among the full fat mealworm powders. The foams of the defatted powders, and especially those of the fermented powders, were significantly (*p* < 0.05) more stable than the foams of the full fat powders and the reference (±74%). If present, oil generally collects at the air-liquid interface and thus interferes with the alignment of the proteins and leads to a decrease in foam stability [24].

The mealworm powders showed superior foaming properties compared with those found in the literature. For example, mealworm flours were reported to have an FC of 32% with an FS of 28% after 30 min [10]. Kim et al. [9] reported an FC of 130% and an FS of 78% (30 min) for water-soluble proteins extracted from defatted mealworm flours. However, as the results differ in method of determination and calculation, they are difficult to compare.

#### 3.2.3. Emulsion Capacity and Stability

To evaluate the emulsifying properties of the different mealworm powders, the emulsion capacity (EC) and the emulsion stability (ES) were measured. The results, presented in Figure 3C,D, show that both parameters were significantly (*p* < 0.05) affected by the fermentation process. Without defatting, the EC decreased from 51% to 5% and 7% upon blanching and fermentation with the starters Bactoferm^®^ F-LC and *L. farciminis*, respectively, while the ES decreased from 74% to 0% and 18%. Reduction in emulsifying properties by fermentation was observed earlier by Lampart-Szczapa et al. [30] for lupin proteins. In this study, the lactic acid fermented lupin proteins were characterised by a lower hydrophobicity than non-modified lupin proteins and, consequently, by worse emulsifying properties. The emulsification properties of potato flours [31] and sorghum flour [32], on the other hand, were improved by fermentation. Their soluble protein concentrations were increased during fermentation, which promotes oil droplet entrapment. Defatting significantly (*p* < 0.05) improved the EC of the fermented powders with 31% and 42% for FLC and Far, respectively, whereas the EC of the Control was unaffected. The ES was either improved (d-Control) or deteriorated (d-FLC and d-Far) by defatting.

The EC and ES of non-fermented mealworm powder are in line with those of other studies on mealworm flours [9,33]. In addition, their emulsification properties were not significantly different from commercial egg albumen powder, indicating its potential as an alternative source of protein emulsifier for food formulations.

#### 3.2.4. pH Dependent Protein Solubility

**Figure 4:**
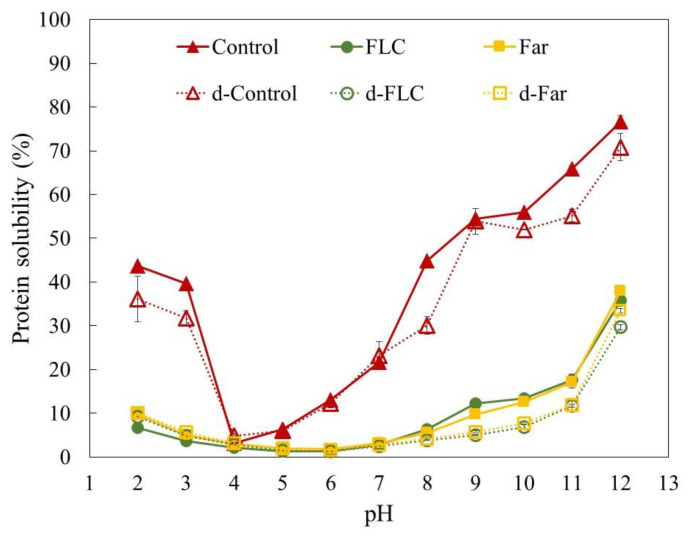
Protein solubility of the full fat (Control and the fermented samples FLC and FAR) and defatted (d-Control and the fermented samples d-FLC and d-Far) mealworm powders as a function of pH. Protein solubility [%] is presented as the total protein content analysed via Kjeldahl method.

Figure 4 shows the protein solubility profiles of the mealworm powders in the pH range of 2 to 12. The protein solubility of the Control decreased in the pH range 2 to 4, showed a minimum solubility of 3% at pH 4, and gradually increased in the pH range 5 to 12. The highest solubility was found at pH 12 (77%). Using similar assay conditions, Bußler et al. [8] and Zielińska et al. [10] reported a maximum protein solubility of respectively 70% at pH 10 and 97% at pH 11. In contrast, less than 3% of the proteins were soluble near the isoelectric point (pH 4–5). Defatting of the Control did not lead to an increased yield of soluble proteins. On the contrary, protein solubility was significantly decreased at low pH (2–3) and high pH (8, 10–12) values. Regardless of the starter culture used, blanching and fermentation led to a drastic decrease in protein solubility and shifted the isoelectric point from pH 4 to pH 6. Similar results were obtained during the fermentation of sorghum by Elkhalifa, Schiffler, and Bernhardt [34]. In this study, lactic acid fermentation shifted the isoelectric point of sorghum proteins by two pH units and decreased the protein solubility due to the exposure of hydrophobic groups.

Loss in solubility indicated denaturation or other structural changes of the mealworm proteins during processing. Both starter cultures tested in this study produced organic acids during fermentation as indicated by the pH reduction, which might have induced an irreversible coagulation of the proteins and thus a reduced solubility [35]. Further, the fermentation process including the blanching step as pre-treatment might have promoted aggregation and cross-linking of partially hydrolysed mealworms proteins, causing them to become insoluble [36]. In this context two molecular aspects need to be considered: on the one side, due to partial hydrolysis occurring during the fermentation, the hydrophobic core of the proteins becomes exposed giving opportunity for aggregation of proteins based on non-covalent interactions. On the other side, the processing combined with fermentation generally leads to disulphide exchange between the exposed cysteine side chains, thus eventually promoting inter- and intramolecular covalent reactions. These two aspects promote the insolubility of the degradation products of the proteins.

#### 3.2.5. Quantification of Free Amino Groups and Protein Molecular Weight Distribution

The amount of free amino groups found via the OPA assay in the protein extracts from the non-fermented powder d-Control (13.31 ± 2.19 mM/g soluble protein) was significantly (*p* < 0.05) lower than those of the fermented powders d-FLC (130.13 ± 2.06 mM/g soluble protein) and d-Far (144.49 ± 18.76 mM/g soluble protein). The high amount of free amino groups in the fermented samples may be attributed to proteolytic degradation of proteins during fermentation. Degradation of proteins resulting in an increase in free amino groups has been detected in many fermented products, such as yoghurt [37], Suanyu (fermented fish, [38]) and mao-tofu (fermented soybean, [39]). The observed increase in free amino groups may point towards a corresponding increase in the free carboxyl groups resulting from the enzymatic degradation of the peptide bonds. Both these observations implicate the possibility of stronger ionic interactions, supporting the formation of non-covalent bonds during the aggregation discussed above, and thus making these molecular forms insoluble over a broader pH range.

**Figure 5:**
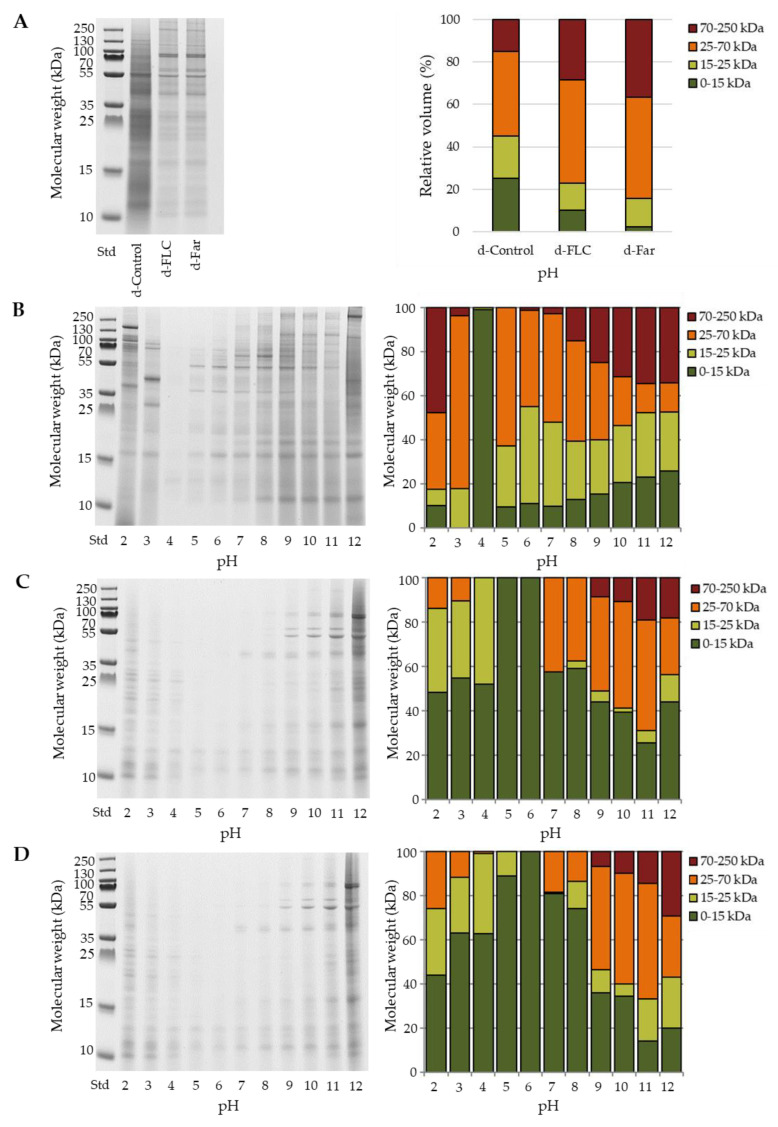
Gel electrophoresis (left) and relative composition (right) of mealworm powders (**A**) and soluble mealworm protein fractions (Control: regular mealworm paste (**B**), d-FLC: mealworm paste fermented with the starter Bactoferm^®^ F-LC (**C**), and d-Far: mealworm paste fermented with the starter *L. farciminis* (**D**) at different pHs. Proteins are classified in four groups: Low molecular weight (0–15 kDa), medium molecular weight (15–25 kDa and 25–75 kDa), and high molecular weight (70–250 kDa).

SDS-PAGE analysis of the mealworm powders (Figure 5A, left figure) showed proteins with molecule weights between 10 and 250 kDa. Bußler et al. [8] already described the protein bands observed in *T. molitor* flour and bands between 32 to 95 kDa appear to be related to enzymes and other proteins, e.g., melanisation-inhibiting protein (43 kDa), β-glycosidase (59 kDa) and trypsin-like proteinases (59 kDa). As to the mealworm powders in Figure 5A, in the non-fermented Control, a band can be seen at about 55 kDa, which is also visible in the fermented samples, and it can be the same type of protein. In the fermented samples, there is an additional clear band between 70 and 100 kDa that may originate from mealworm proteins, such as melanisation-engaging types of protein (85 kDa), but as the band is not present in the non-fermented Control, it cannot be excluded that it originates from the starter cultures. Further identification of the bands is very difficult based on the data available. As can be seen in Figure 5A (right figure) and contrary to what can be expected, in relative terms fermented mealworm powders were more abundant in medium (25–70 kDa) and high (70–250 kDa) molecular weight proteins and less abundant in low-molecular weight proteins (0–25 kDa) than the non-fermented powders. This relative increase in medium and high molecular weight proteins by the blanching plus fermentation process is probably explainable by the fact that (1) during fermentation some of the low-molecular weight proteins are consumed by the starter cultures to ensure their growth (and as a result the relative abundances of medium/high molecular weight proteins increases) and/or that (2) the proteins with a molecular weight of 70 kDa or higher originate from the added starter culture cells. A further contribution to this observation may be related to macro protein structures that in the insects as such were not soluble but become more accessible after the fermentation due to partial hydrolysis. Further in-depth analysis is needed to characterize or identify the origin of these proteins and is envisaged in further studies. SDS-PAGE analysis of the soluble protein content of the non-fermented and fermented mealworm powders revealed that the fermentation process leads to a shift towards lower molecular weights of the proteins and that, depending on the pH, different protein fractions are soluble (Figure 5B–D). At pH 7, which was used for the evaluation of the emulsifying and foaming properties, the protein extract of the non-fermented mealworm powder was composed of 2.7% high molecular weight fraction, 72.1% medium molecular weight fraction, and 9.8% low molecular weight fraction, whereas the protein fractions characterized by low molecular weights (0–15 kDa) were found to dominate the protein extracts of the fermented mealworm powders (57.4% and 80.9% for d-FLC and d-Far, respectively). As the molecular sizes of the soluble proteins decreased, it was clear that also the protein structure was modified by the fermentation process, which in turn led to changes in techno-functional properties. Rahali et al. [40] and Razali et al. [41] reported that the surface hydrophobicity of the proteins is more important than the peptide length in emulsion and foaming properties. Most often, high surface hydrophobicity is needed to allow the formation of stable emulsions and foams. The hydrophilic/hydrophobic character of proteins is connected to their secondary, tertiary, and quaternary structure and is caused by the amphiphilic character of amino acids and their accessibility in the polypeptide chain [42]. It could be hypothesized that the low molecular weight peptides produced by fermentation can migrate rapidly to the interface but that their hydrophilic/hydrophobic balance was insufficient for the stabilization of emulsions and foams.

## 4. Conclusions

Previous research has shown that fermentation of mealworm paste with lactic acid starter cultures results in a rapid pH reduction, as an indication of a successful fermentation process. In this study, the effect of blanching and fermentation on the functional properties was considered. When after the fermentation the flour is not defatted, WBC and OBC are decreased, the FC and FS are not affected, and the EC and ES are reduced. When after the fermentation the flour is defatted, the same effects can be seen, with the exception that WBC and FS are (somewhat) improved compared to the non-fermented powders. The differences in protein functionality between non-fermented and fermented mealworm powders may be ascribed to differences in the proportions of the different ingredients of the powders as indicated by the crude protein and crude fat analysis, differences in the molecular size of the proteins as indicated by the analysis of the protein distribution, and probably due to differences in the hydrophilic/hydrophobic arrangement. Overall, it can be concluded that fermentation of mealworm paste with the starter cultures Bactoferm^®^ F-LC and *L. farciminis* does not contribute to the functional properties studied in this work. Therefore, fermentation with these starter cultures can rather be investigated as a technology for taste and shelf life improvement.

## Figures and Tables

**Table 1:** Means ± standard deviations (*n* = 3) of dry matter (DM), crude protein (CP), crude fat content (CF), pH, bulk density (BD), browning indices (BI) and total colour difference with the control sample (ΔE) of full fat (Control and the samples fermented with Bactoferm FLC and *Lactobacillus farciminis*, FLC and Far) and defatted powders (d-Control and the fermented samples d-FLC and d-Far) produced from non-fermented and fermented mealworm pastes.

Powder	DM [g/100g as is]	CP [g/100 g DM]	CF [g/100 g DM]	pH [-]	BD [g/mL]	BI [-]	ΔE ^1^
Control	96.26 ± 0.43 ^c^	49.68 ± 0.02 ^c^	16.61 ± 0.23 ^c^	6.28 ± 0.05 ^d^	0.35 ± 0.00 ^a^	64.77 ± 0.13 ^b^	-
FLC	95.76 ± 0.13 ^c^	42.60 ± 0.02 ^a^	21.05 ± 0.43 ^e^	4.60 ± 0.04 ^a^	0.40 ± 0.01 ^b,c^	72.30 ± 0.42 ^d^	2.35 ± 0.04 ^a^
Far	96.08 ± 0.10 ^c^	44.55 ± 0.04 ^b^	20.20 ± 0.31 ^d^	4.64 ± 0,04 ^a,b^	0.41 ± 0.01 ^c^	68.24 ± 1.08 ^c^	2.16 ± 0.19 ^a^
d-Control	94.35 ± 0.21 ^b^	67.89 ± 0.02 ^e^	3.37 ± 0.10 ^a^	6.28 ± 0.02 ^d^	0.39 ± 0.01	28.17 ± 0.78 ^a^	-
d-FLC	92.19 ± 0.07 ^a^	61.94 ± 0.02 ^d^	5.13 ± 0.21 ^b^	4.72 ± 0.02 ^b,c^	0.49 ± 0.01 ^d^	28.11 ± 2.45 ^a^	1.76 ± 0.60 ^a^
d-Far	92.21 ± 0.07 ^a^	61.86 ± 0.04 ^d^	3.17 ± 0.15 ^a^	4.75 ± 0.02 ^c^	0.49 ± 0.01 ^d^	30.25 ± 0.50 ^a^	1.97 ± 0.58 ^a^

^1^ The colour of full fat fermented samples was compared to the full fat control (Control) and the colour of defatted fermented samples was compared to the defatted control (d-Control). ^a,b,c,d,e^ Mean values within a column with the same superscript are not statistically different (*p* > 0.05).

## Supplementary Materials

The following is available online at https://www.mdpi.com/2304-8158/9/7/917/s1, Table S1: Initial protein concentration, quantity of protein loaded on the gel and relative and absolute abundances of the soluble proteins with molecular weight 0–15 kDA of non-fermented (d-Control) and fermented (d-FAR and d-FLC) mealworm powders.
