# Supplementary material for: Effect of Blanching Plus Fermentation on Selected Functional Properties of Mealworm (Tenebrio molitor) Powders
Source: Foods. 2020 Jul 12;9(7):917. doi: 10.3390/foods9070917 (PMC7405005; doi:10.3390/foods9070917)
Supplement: Supplementary file 1 [file foods-09-00917-s001.pdf]

Table S1. Initial protein concentration, quantity of protein loaded on the gel and relative and absolute abundances of the soluble proteins with molecular weight 0-15 kDA of non-fermented (d-Control) and fermented (d-FAR and d-FLC) mealworm powders.

Well No	1	2	3	4	5	6	7	8	9	10	11	12
Sample	Std	pH 2	pH 3	pH 4	pH 5	pH 6	pH 7	pH 8	pH 9	pH 10	pH 11	pH 12
**D-Control**												
Initial protein concentration (µg/ml)	/	2097.1	2028.6	314.3	387.5	780.4	1480.1	1899.1	3448.9	3317.3	3479.8	4494.7
Quantity of protein loaded (µg)	/	6.29	6.09	0.94	1.16	2.34	4.44	5.7	10.35	9.95	10.44	13.48
MW 0-15 kDA (relative)		14	0	96.5	8.14	15.11	13.95	16.28	18.60	24.41	26.74	29.07
MW 0-15 kDA (absolute)		0.88	0	0.97	0.09	0.35	0.62	0.93	1.92	2.43	2.79	3.91
**d-FLC**												
Initial protein concentration (µg/ml)	/	575.3	267.9	162.4	81.7	78.7	142.5	225.3	284.3	387.8	653.4	1677.4
Quantity of protein loaded (µg)	/	1.78	0.89	0.54	0.27	0.26	0.48	0.75	0.95	1.29	2.18	5.59
MW 0-15 kDA (relative)		52.38	57.14	54.76	100	100	60.71	61.90	47.62	42.86	29.76	47.62
MW 0-15 kDA (absolute)		0.93	0.51	0.30	0.27	0.26	0.29	0.46	0.45	0.55	0.64	2.66
**d-Far**												
Initial protein concentration (µg/ml)	/	533.4	323.3	181.5	116.2	96.7	156.2	233.6	320.6	445.2	683	1884.1
Quantity of protein loaded (µg)	/	1.92	1.08	0.61	0.39	0.32	0.52	0.78	1.07	1.48	2.28	6.28
MW 0-15 kDA (relative)		47.06	63.53	63.53	89.41	100	81.18	75.29	40	37.65	16.47	23.3
MW 0-15 kDA (absolute)		0.61	0.69	0.39	0.35	0.32	0.42	0.59	0.43	0.56	0.38	1.46
